# COVID-19 infection in infant with severe CHD

**DOI:** 10.1017/S1047951121001384

**Published:** 2021-03-18

**Authors:** Natalie Pexton, Amy Svenson, Deepti Bhat

**Affiliations:** Department of Cardiology, Phoenix Children’s Hospital, 1919 Thomas Rd, Phoenix, AZ 85016, USA

**Keywords:** COVID-19, SARS-COV-2, hypoplastic left heart syndrome

## Abstract

We describe the case of a 2-month-old born with hypoplastic left heart syndrome who presented with fever and vomiting and was found to be infected with the novel corona virus (COVID-19). He underwent treatment with supplemental oxygen, heparin, and dexamethasone. After a 6-day hospitalisation, he recovered remarkably well without major adverse effects.

The novel coronavirus, responsible for the current worldwide pandemic, appears to cause multi-system disease with high morbidity and mortality in patients with comorbid conditions. Current data suggest children tend to be low risk for serious illness. However, infants with single-ventricle physiology form a medically fragile population and are at high risk for morbidity and mortality from any intercurrent illness.^1–3^ We present the case of a 2-month-old infant born with hypoplastic left heart syndrome and his clinical course after he contracted the novel corona virus.

## Clinical course

This is a 2-month-old born with hypoplastic left heart syndrome (mitral and aortic atresia) who underwent Stage 1 palliative surgery with Norwood-Sano approach. He had been discharged home after a prolonged post-operative recovery and was closely followed by our interstage single-ventricle team. He presented to the emergency room with 1 day of fever and vomiting. Parents reported home saturations within the target range of 75–85% on home pulse oximeter. History revealed his father was ill and later tested positive for the novel corona virus. The patient’s vital signs in the emergency room were significant for tachycardia (169 beats/minute), fever (38.7°C), tachypnea (65 breaths/minute), and SpO2 77%. He was ill appearing on examination with poor perfusion when crying.

His venous blood gas analysis was significant for elevated lactic acid (4.7 mEq/L) with metabolic acidosis (pH 7.33/pCO2 37 mmHg/HCO3 20 mmol/L). Chest X-ray showed clear lungs and no change in size of the cardiac silhouette. Standard viral panel was negative and severe acute respiratory syndrome coronavirus 2 (SARS-CoV-2) ribonucleic acid was positive.

He was started on 1 L supplemental oxygen by nasal cannula and admitted to the ICU. On hospital day 2, he had recurrent fever to 38.4°C. The patient continued to have desaturations, requiring increase in FiO2 to 50%. He was placed on heated humidified nasal cannula (6 litres/minute and FiO2 30%). His home dosing of furosemide was held only on hospital day 1. Home dosing of aspirin and amiodarone was continued. Repeat venous blood gas demonstrated interval improvement with lactic acid down from 4.7 to 2.9 mEq/L. Echocardiogram on hospital day 2 showed no dilation or ectasia of the coronary arteries but showed slightly decreased single right ventricular systolic function from “normal” to “mildly decreased” by change in fractional area and slight worsening of the tricuspid valve regurgitation from “mild” to “mild to moderate” by jet size.

Coagulation studies were reassuring. However, given the patient’s worsening clinical status and high-risk shunt-dependent circulation, he was empirically placed on low molecular weight heparin infusion of 20 units/kg/hour IV on hospital day 3 with a goal Anti-Xa level of 0.1–0.3 U/ml to prevent thrombotic phenomena.^4,5^ Dexamethasone IV 0.75 mg was also initiated and given once a day for 5 days.^6^

By hospital day 3, the patient was afebrile, weaned to room air with saturations >75%. On hospital day 4, oxygen saturations were trending down to the high 60 s on room air with a haemoglobin of 12.1 gm/dL. He underwent two transfusions totalling 25 mL/kg packed red blood cell over the next 2 days. Saturations improved to the high 70 s, with follow-up haemoglobin of 17.9 gm/dl. Heparin was stopped and he was discharged home on room air on hospital day 6.

Following hospital discharge, he had no recurrence of fever, desaturations, or respiratory distress. His follow-up echocardiogram 1 month later demonstrated persistent mildly diminished right ventricular function and mild-to-moderate tricuspid valve regurgitation. Six weeks following his hospitalisation for the novel corona virus, he underwent routine cardiac catheterisation in preparation for the second staged surgery. The Qp to Qs was 0.93 demonstrating the patient’s lung and systemic circulation were relatively well balanced. Pressure measurements were suggestive of low pulmonary vascular resistance.

## Discussion

Our patient did remarkably well, recovering after 6 days from a novel corona virus infection with a mild decrease in cardiac function and tricuspid regurgitation by echocardiogram. The cardiac changes may be related to tachycardia causing diminished coronary artery profusion during the illness period or secondary to his underlying CHD.

Coagulopathy has been widely reported in patients with novel corona virus. A study by Tang et al out of Wuhan showed 449 patients with severe novel corona virus and high sepsis-induced coagulopathy score or elevated D-dimer had decreased mortality with heparin administration.^4,5^ We considered our shunt-dependent patient high risk for thrombotic events. Thus, we treated empirically with low molecular weight heparin therapy in addition to home aspirin. The decision to administer dexamethasone was based on the RECOVERY trial, which showed that amongst 6000 adults with novel corona virus, patients who received dexamethasone had decreased mortality (by about 20%) compared with those who did not.^6^

## Conclusion

In theory, our patient with single-ventricle physiology is high risk for serious illness from novel corona virus. It is unknown whether our patient had a milder illness or the outcome was due to early diagnosis and treatment. This case demonstrates it is possible for these children to survive without major adverse effects from the initial illness. While this case is not predictive for other patients with CHD presenting with novel corona virus, the clinical course may be useful to others caring for this population.

Due to lack of current knowledge about the long-term consequences of novel corona virus on children and the CHD population, children with CHD who become infected with the virus should continue to be followed by cardiology.
